# Treatment of *Mycobacterium abscessus* subsp. *massiliense* Tricuspid Valve Endocarditis

**DOI:** 10.3201/eid2103.140577

**Published:** 2015-03

**Authors:** R. Gordon Huth, Elizabeth Douglass, Kristin Mondy, Sruthi Vasireddy, Richard J. Wallace

**Affiliations:** The University of Texas at Austin, Dell Medical School, Austin, Texas, USA (R.G. Huth, E. Douglass, K. Mondy);; University of Texas Health Science Center, Tyler, Texas, USA (S. Vasireddy, R.J. Wallace Jr.)

**Keywords:** nontuberculous mycobacteria, endocarditis, Mycobacterium abscessus, massiliense, tuberculosis and other mycobacteria, bacteria, tricuspid valve endocarditis

**To the Editor:** *Mycobacterium abscessus* is a ubiquitous, rapidly growing mycobacteria (RGM) found in water supplies, soil, and dust. *M. abscessus* is considered the most pathogenic and difficult to treat of the RGM and is most often associated with pulmonary, skin, and soft tissue infections; it has also been reported to cause ocular infections, otitis, lymphadenitis, arthritis, osteomyelitis, disseminated disease, and prosthetic valve endocarditis (*1*,*2*). Most prosthetic valve endocarditis cases have been fatal.

*M. abscessus* subsp. *massiliense* is 1 of 3 subspecies of *M. abscessus*. *M. abscessus* subsp. *massiliense* has an identical 16S rRNA gene sequence to the other 2 subspecies, *Mycobacterium abscessus* subsp. *bolletii* and *Mycobacterium abscessus* subsp. *abscessus*, but can be differentiated by *rpo*β and *erm41* gene sequencing (*3*,*4*). *M. abscessus* subsp. *massiliense* grows readily in blood culture media and on sheep’s blood agar within 2–4 days. Care should be taken in interpreting Gram staining of isolates because RGM is not identifiable by this method and could be mistaken for corynebacteria or diphtheroids (*5*,*6*). Such isolates could be further tested by acid-fast staining and, if positive, sent to a reference laboratory for definitive identification and susceptibility testing.

Five cases of *M. abscessus* native valve endocarditis have been reported; 4 were fatal and 1 was lost to follow-up (*1*,*5*–*9*). One of the 4 fatal cases also involved the tricuspid valve and was associated with intravenous heroin abuse (*9*). We report a case of *M. abscessus* subsp. *massiliense* native tricuspid valve endocarditis successfully treated with antimicrobial therapy and surgical debridement.

A 52-year-old man who used intravenous drugs was admitted to our hospital describing a 25-pound weight loss, fever, and night sweats. He reported injecting crushed opioid tablets mixed with tap water. He had tachycardia and pitting edema of the legs and feet. Laboratory data revealed elevated minotransferase levels, thrombocytopenia, and opiates in the urine. Computerized chest tomographic scan showed cavitary right upper lobe and lingular nodules. Routine blood cultures (BacT/ALERT3D; bioMérieux, Marcy l’Etoile, France) of samples drawn at admission and on hospital day 3, before the initiation of antimicrobial drug therapy, grew acid-fast bacilli (AFB) in broth medium on days 3 and 4 of incubation. A transthoracic echocardiogram on hospital day 5 revealed a 1-cm vegetation on the tricuspid valve. An empiric regimen for RGM consisting of intravenous cefoxitin and amikacin and oral clarithromycin and moxifloxacin were administered. Based on preliminary (3-day) susceptibility test results showing susceptibility to amikacin, resistance to the quinolones, and intermediate susceptibility to cefoxitin, linezolid, and imipenem, the regimen was changed to tigecycline, linezolid, clarithromycin, and amikacin (*10*). Routine blood cultures on hospital days 11 and 17 were negative.

On hospital day 19, linezolid was stopped, and imipenem was added. A transthoracic echocardiogram on hospital day 31 showed the vegetation had enlarged to 1.5 × 0.5 cm. We concluded that antibiotics alone were unlikely to be curative; cardiac catheterization was performed on hospital day 38. On the basis of hemodynamic findings, the cardiologist inferred that valve replacement would be of no value and recommended valvectomy alone.

Surgery on hospital day 41 revealed a 2-cm nodule on each anterior and posterior leaflet and a 2–3 mm nodule on the septal leaflet of the tricuspid valve. The anterior and posterior leaflets were removed, and the septal leaflet was segmentally resected. Routine cultures of valve tissues, in which *M. abscessus* would have grown, were negative. Pathologic examination confirmed suppurative vegetations with numerous bacterial colonies consistent with AFB; AFB staining disclosed numerous mycobacteria.

Identification and final susceptibilities of the RGM from the original blood culture isolate revealed *M. abscessus* subsp. *massiliense* by *hsp65* PCR and *erm* gene sequencing (*4*) and 14-day susceptibility to clarithromycin (*10*). *M. abscessus* subsp. *massiliense* has a nonfunctional (truncated) macrolide-inactivating gene (*erm41*), and untreated isolates are susceptible to the macrolides (*4*).

Repeat chest tomographic scan on hospital day 69 showed nearly complete resolution of the RUL cavitary and lingular nodules/infiltrates. Tigecycline, amikacin, imipenem, and clarithromycin were continued until hospital day 77, when amikacin was stopped because of moderate hearing loss. The patient was discharged without antibiotics after 2 months of postoperative antibiotic therapy. At follow-up visits 2 and 8 weeks later he was doing well except for peripheral edema. AFB and routine blood cultures drawn at both visits were negative. He is periodically seen in the cardiology clinic; his edema has resolved with diuretic therapy.

Cure of *M. abscessus* native valve endocarditis has not been previously reported. A case of *M. chelonae* native tricuspid valve endocarditis associated with a pacemaker lead was successfully treated with wire removal, valve debridement, and antimicrobial therapy (*11*). The patient in the current study likely acquired his infection from the tap water diluent he injected. Clinicians should consider the possibility of mycobacterial endocarditis when evaluating a septic patient with intravenous drug use history or cardiac prosthetic devices.

We successfully treated mycobacterial native tricuspid valve endocarditis with combination antimicrobial therapy and surgical debridement. The location of the infection in the tricuspid valve and favorable hemodynamics enabled debridement without implantation and the subsequent possibility of intraoperative infection of a prosthetic valve.
